# Distinct Roles of the Premotor and Occipitotemporal Cortices in the Full-Body Illusion

**DOI:** 10.1523/ENEURO.0587-24.2025

**Published:** 2025-09-26

**Authors:** Katsuki Higo, Itsuki Ohtsuka, Sotaro Shimada

**Affiliations:** ^1^ Organization for the Strategic Coordination of Research and Intellectual Properties, Meiji University, Kanagawa 214-8571, Japan; ^2^ Graduate School of Science and Technology, Meiji University, Kanagawa 214-8571, Japan; ^3^School of Science and Technology, Meiji University, Kanagawa 214-8571, Japan

**Keywords:** embodiment, full-body illusion, multisensory integration, near-infrared spectroscopy, self-recognition, sense of body ownership

## Abstract

The sense of body ownership, a core aspect of self-recognition, has been studied using illusions such as the full-body illusion. Although the premotor cortex is considered central to body ownership in first-person full–body illusions, the occipitotemporal cortex—including the temporoparietal junction (TPJ) and the extrastriate body area (EBA)—also plays a critical role in third-person full–body illusions. However, their distinct contributions to the full-body illusion remain unclear, partly due to the challenges of applying neuroimaging in such experiments. This study employed functional near-infrared spectroscopy to investigate brain activity during a third-person full–body illusion in virtual reality. Eighteen healthy human adult males participated in the study. The experiment consisted of two sessions. In Session 1, participants observed an avatar's back receiving either synchronous or asynchronous visual–tactile stimulation. In Session 2, visual stimuli alone were presented to participants after they experienced the full-body illusion to induce visuotactile discrepancies. In the synchronous condition of Session 1, we found significant deactivation in the superior and middle temporal gyri (partially including the TPJ), followed by higher activity than in the asynchronous condition in the left middle occipital gyrus (likely EBA). The left premotor cortex also showed significant activation (uncorrected), although this did not survive multiple-comparison adjustment. In Session 2, the visuotactile discrepancy induced significant left premotor activation only in the synchronous condition (FDR-corrected). These findings suggest that the occipitotemporal cortex supports receptivity to third-person full–body illusions, whereas the premotor cortex contributes to maintaining illusory body ownership by reconciling multisensory conflicts.

## Significance Statement

The relative importance of the premotor cortex versus the occipitotemporal cortex—including the temporoparietal junction (TPJ) and the extrastriate body area (EBA)—in body ownership remains debated. To address this, we used functional near-infrared spectroscopy to measure brain activity during full-body illusion. Our findings revealed significant deactivation in the TPJ, followed by greater EBA activation in the illusion-induced condition than in the no-illusion condition, indicating the occipitotemporal cortex establishes receptivity to third-person full–body illusions. Furthermore, increased activity in the premotor cortex during sensory conflict may reflect its involvement in resolving multisensory conflicts and sustaining body ownership of the avatar. These results offer insights into the neural mechanisms underlying body ownership in the full-body illusion.

## Introduction

The sense of body ownership, which is crucial for self-recognition, is often studied using the full-body illusion (Lenggenhager et al., 2007; Petkova and Ehrsson, 2008), in which a fake body is perceived as one's own through synchronous visual and tactile stimulation applied to the fake and real bodies. However, the neural mechanisms responsible for the full-body illusion have not been fully investigated, mainly due to difficulties in measuring brain activity while participants are standing or wearing a head-mounted display (HMD), which are usually employed in the full-body illusion experiments.

Despite this, several studies have reported brain activity during the full-body illusion, which used functional magnetic resonance imaging (fMRI; Petkova et al., 2011a; Gentile et al., 2015; Guterstam et al., 2015a,b; Preston and Ehrsson, 2016; see also Ehrsson, 2020, for review). In these studies, participants observed a fake body from a first-person perspective (the “body-swap” type), with the fake body's parts stroked synchronously with the participant's to elicit the full-body illusion. The results revealed the involvement of brain regions similar to those reported in earlier rubber hand illusion studies (Ehrsson et al., 2004; Tsakiris, 2010; Brozzoli et al., 2012; Ehrsson, 2012; Gentile et al., 2013; Blanke et al., 2015). In particular, the premotor cortex is involved in generating the sense of body ownership (Guterstam et al., 2015a,b; Preston and Ehrsson, 2016) and plays a principal role in integrating various body parts—such as the abdomen, arms, and legs—into a coherent whole-body representation (Petkova et al., 2011a; Gentile et al., 2015).

Meanwhile, other studies have highlighted the role of the temporoparietal junction (TPJ) and the lateral occipital cortex—especially the extrastriate body area (EBA)—in the full-body illusion induced from a third-person perspective (the “out-of-body” type). Ionta et al. (2011) studied neural activity using fMRI, in which participants were shown the back of another person's body being stroked with a rod. The TPJ showed activity corresponding to changes in self-location caused by the illusion, which aligns with reports that electrical stimulation of the TPJ leads to out-of-body experiences (Blanke et al., 2002). Previous studies also demonstrated that the EBA is critically involved in the rubber hand illusion (Wold et al., 2014; Limanowski and Blankenburg, 2015, 2016). These findings suggest that both the TPJ and EBA contribute to the sense of ownership of the body. However, their precise roles remain under debate. Some researchers argue that the EBA primarily processes visual bodily information (Downing et al., 2001; Makin et al., 2007; Blanke et al., 2010; Heydrich and Blanke, 2013; Perruchoud et al., 2016), while others propose that it contributes to the multisensory processes underlying body ownership (Wold et al., 2014; Limanowski and Blankenburg, 2016).

Thus, there are two perspectives regarding the brain functions involved in the full-body illusion: one postulates that the premotor cortex is crucial, while the other emphasizes the importance of the TPJ and EBA. Therefore, this study aims to investigate the distinct roles of the premotor cortex, TPJ, and EBA during the full-body illusion. We employed a virtual reality (VR) environment in which the participant observed an avatar standing in a virtual space while brain activity was measured using functional near-infrared spectroscopy (fNIRS). Unlike previous studies using fMRI, where participants were measured in a supine position in the scanner, our study recorded brain activity in an upright posture using fNIRS. This distinction is important, as the sense of body ownership is known to be influenced by gravity and equilibrium (Ionta et al., 2011; Preuss and Ehrsson, 2019). Our approach offers a more ecologically valid investigation of the neural mechanisms underlying the full-body illusion.

This experiment comprises two sessions. In Session 1, synchronous or asynchronous visuotactile stroking was applied to the avatar and the participant to examine brain activity associated with the embodiment of the avatar. In Session 2, only visual stroking (with no tactile stimulation applied to the participant) was applied to the avatar, thereby introducing a visuotactile discrepancy. Prior research suggests that the motor area, including the premotor cortex, is crucial for multisensory integration in body ownership and also plays an important role in resolving multisensory conflicts (Schutz-Bosbach et al., 2006; Lee and Chae, 2016; Shibuya et al., 2018; Shimada, 2022). These studies show that visuo–proprioceptive or visuotactile discrepancies activate the motor area. We hypothesize that the synchronous condition in Session 1 induces brain activity associated with avatar embodiment, while Session 2 will reveal neural activity related to conflict resolution, particularly in the premotor cortex.

## Materials and Methods

### Participants

Eighteen healthy students (all male; age 21.6 ± 1.0 years SD) were recruited for this experiment. All participants provided written informed consent before the experiment. This study was approved by the Ethics Committee of the School of Science and Technology, Meiji University.

### Apparatus

**Figure 1.**
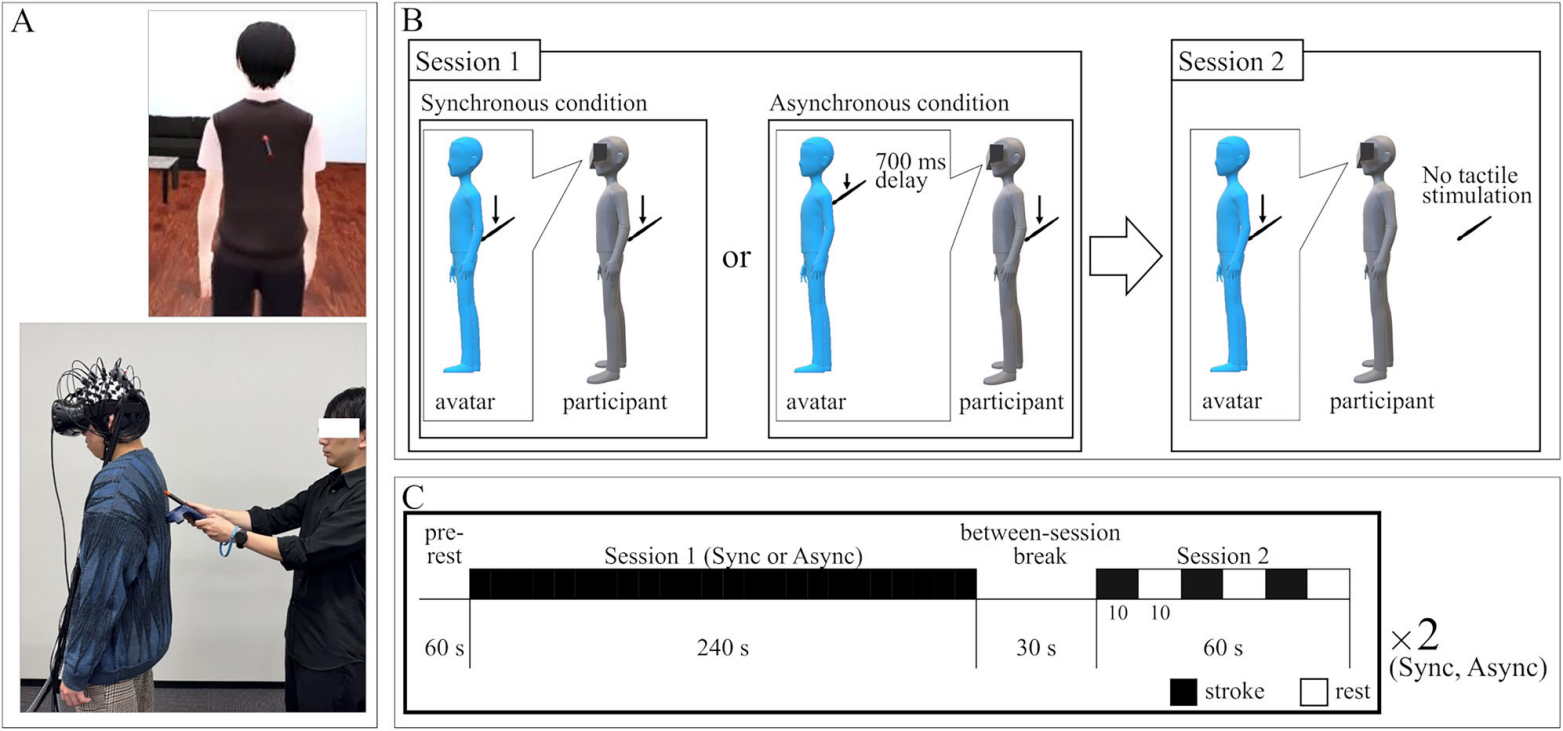
The VR environment and experimental procedure. ***A***, Participants viewed the back of a virtual avatar. The avatar and the participant were stroked on their backs. ***B***, The experiment consisted of two sessions. In Session 1, the avatar's and participant's backs were stroked. In the synchronous condition, stroking was temporally aligned; in the asynchronous condition, the avatar’s stroking was delayed by 700 ms. Session 2 presented only visual stimulation (stroking of the avatar) without tactile input to the participant. The condition label (synchronous or asynchronous) in Session 2 corresponded to the type of visuotactile stimulation previously applied in Session 1. ***C***, Session 1 lasted 240 s, and Session 2 lasted 60 s. Session 2 consisted of three repetitions of 10 s of stroke followed by 10 s of rest.

An HMD (HTC Vive, HTC; 2,160 × 1,200 resolution; 110° diagonal field of view; refresh rate, 90 Hz) was used to display the VR environment to the participants. HTC Vive Trackers (HTC) were mounted on the backs of both hands, insteps, and waist to reflect the participant's movements in the avatar. The experimental VR environment was developed using the Unity 2019.2.2f1 software platform (Unity Technologies; Fig. 1*A*).

### Procedure

This experiment consisted of two experimental conditions—synchronous and asynchronous—each comprising two sessions (Session 1 and Session 2; Fig. 1*B,C*). In Session 1, we investigated brain activity during the induction of a full-body illusion in a VR space by presenting either synchronous or asynchronous visuotactile stimulation (i.e., visual and tactile stroking). A 1 min prerest period was included at the beginning of Session 1 to obtain baseline brain activity. Session 2 assessed the neural response to purely visual stimulation (i.e., stroking of the avatar's back without tactile input), following the visuotactile stimulation in Session 1. Importantly, the condition label (synchronous or asynchronous) for Session 2 was determined by the type of stimulation received in the preceding Session 1. That is, if Session 2 followed synchronous (or asynchronous) visuotactile stimulation in Session 1, it was labeled as the synchronous (or asynchronous) condition accordingly. The procedure during Session 2 was identical across both conditions. A 30 s break separated Session 1 and Session 2. We employed a within-subject design, with the order of synchronous and asynchronous conditions counterbalanced across participants.

During the experiment, participants observed the back of a VR avatar. In Session 1, a tactile stimulus was applied by the experimenter stroking the participant's back with a stick, while a visual stimulus showing the avatar's back being stroked was displayed simultaneously for 4 min. The stick was fixed to a VR controller, and its movements were mirrored by a virtual stick in the VR environment. In the synchronous condition, the tactile stimulation on the participant's back and the visual stimulation on the avatar's back were delivered simultaneously. In contrast, in the asynchronous condition, the visual stimulation was delayed by 700 ms relative to the tactile stimulation to induce visuotactile incongruence. Previous studies have shown that a 700 ms delay in visual feedback is sufficient to disrupt the sense of body ownership in self-body illusions (Shimada et al., 2009; Ismail and Shimada, 2016, 2019; Nakul et al., 2020). Accordingly, we employed a unity program to introduce a 700 ms delay in mapping the controller's movements onto the virtual stick. The strokes were delivered at a rate of ∼1 Hz (i.e., one stroke per second).

In Session 2, only the visual stimulation of stroking the avatar's back was presented, without tactile stimulation to the participant's back. In Session 1, the full-body illusion is likely to occur in the synchronous condition; therefore, the visuotactile discrepancy in Session 2 would attenuate the sense of body ownership over the avatar. Conversely, in the asynchronous condition, the discrepancy would not have such an effect, as the sense of body ownership over the avatar would not have been elicited in the first place. The stimulus was presented for 10 s, followed by a 10 s rest; this sequence was repeated three times. We assume that the illusion persisted after the 30 s break following Session 1. This assumption appears reasonable for two main reasons. First, in the rubber hand illusion, subjective ownership has been shown to persist for up to 300 s after the cessation of brush strokes (Abdulkarim et al., 2021). Therefore, it is reasonable to assume that the illusion continued during the 30 s break in this experiment. Second, because the participant's body movements were mapped onto the avatar even during the interval, any spontaneous movements were unlikely to disrupt the illusion. Taken together, these points support the plausibility that the illusion persisted into Session 2. Additionally, throughout the experiment, participants were instructed to stand upright and remain still, and the task did not require any voluntary movement. Thus, interference with the measurement of brain activity was likely minimal.

### Questionnaire

Participants completed a questionnaire on the full-body illusion (Lenggenhager et al., 2007) after each condition in Session 1. They rated their subjective experience on a seven-point Likert scale ranging from −3 (totally disagree) to +3 (totally agree). The questionnaire included seven items: Items 1–3 related to the full-body illusion, and Items 4–7 were dummy items. Table 1 shows the questionnaire items.

**Table 1.** Questionnaire items

Q1	It seemed as if I were feeling the touch of the stick in the location where I saw the virtual body touched
Q2	It seemed as though the touch I felt was caused by the stick touching the virtual body
Q3	It felt as if the virtual body was my body
Q4	It felt as if my (real) body was drifting toward the front (toward the virtual body)
Q5	It seemed as if I might have had more than one body
Q6	It seemed as if the touch I was feeling came from somewhere between my own body and the virtual body
Q7	It appeared (visually) as if the avatar was drifting backward (toward the real body)

### fNIRS data acquisition

We used a multichannel fNIRS unit (OMM-3000, Shimadzu) that employed three wavelengths of near-infrared light (780, 805, and 830 nm). This apparatus allowed us to measure changes in the concentrations of oxygenated hemoglobin (oxyHb), deoxygenated hemoglobin (deoxyHb), and total hemoglobin (totalHb). The sampling rate was set at 10 Hz.

**Figure 2.**
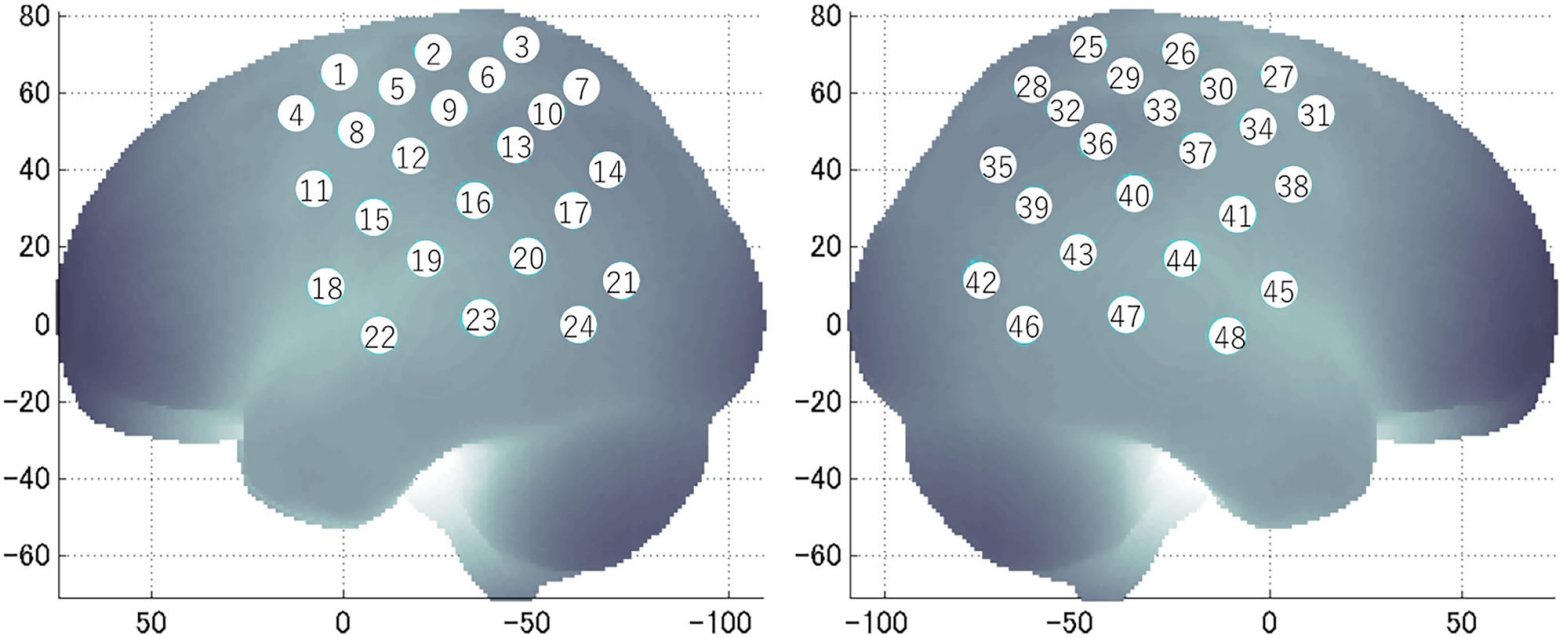
CH locations. NIRS data were recorded from 48 CHs (24 CHs on each side).

We set the fNIRS probes to cover both sides of the brain, including the premotor, lateral occipitotemporal, and parietal cortices. Specifically, we used two sets of 4 × 4 multichannel probe holders, consisting of eight illuminating and eight detecting probes arranged alternately at an interprobe distance of 3 cm, resulting in 24 channels (CHs) per set (Fig. 2). C3/C4 of the 10–20 system was at the center of each probe holder.

We employed spatial registration (Singh et al., 2005) to register the fNIRS data to the standard Montreal Neurological Institute (MNI) brain template. Before the experiment, we measured the relative positions of the probes using a three-dimensional magnetic space digitizer (Fastrak, Polhemus). Based on these data, we estimated the most likely anatomical locations of all probe positions according to anatomical labeling information coded in a macroanatomical brain atlas (LBPA40; Shattuck et al., 2008).

### Statistics

Statistical analyses were performed using R ver. 4.1.1 (R Development Core Team). The Shapiro–Wilk test revealed that the questionnaire scores and fNIRS data were not normally distributed. Therefore, nonparametric methods were used for all statistical analyses.

### Questionnaire

We compared the questionnaire scores between the two conditions using the Wilcoxon signed-rank test. We also applied a one-sample Wilcoxon test against zero to examine the occurrence of the sense of body ownership of the avatar.

### fNIRS data analysis

We analyzed the oxyHb signal as an index of brain activity because it reflects changes in cerebral blood flow accurately and correlates strongly with the BOLD signal among the three signals (oxyHb, deoxyHb, and totalHb) obtained by fNIRS (Hoshi et al., 2001; Strangman et al., 2002). A low-pass filter of 0.3 Hz and a high-pass filter of 0.01 Hz were applied to the signals. Additionally, we employed the hemodynamic modality separation method to remove systemic physiological artifacts (Yamada et al., 2012). Data processing was performed using MATLAB R2021b (MathWorks).

For Session 1, the data were normalized using the mean and standard deviation from the last 30 s of its prerest period. To investigate brain activity during the full-body illusion induction phase (i.e., the 4 min stroking period), we compared the average oxyHb concentration between the two conditions using a sliding-window approach. The sliding window had a length of 60 s and a step size of 5 s. Statistical comparisons of the average oxyHb concentration within each time window between the synchronous and asynchronous conditions were performed using the Wilcoxon signed-rank test.

For Session 2, the data were analyzed using a general linear model (GLM; Friston et al., 1995; Schroeter et al., 2004). A hemodynamic response function was generated by convolving a Gaussian function (4 s full-width at half-maximum) with a boxcar function reflecting the task and rest durations. We compared the *t* values between the two conditions as indices of task-related brain activity using the Wilcoxon signed-rank test. We further employed the one-sample Wilcoxon test against zero to confirm whether there was significant activation or deactivation in areas where a significant difference between the synchronous and asynchronous conditions was found.

We performed statistical analyses on regions of interest (ROIs). We combined neighboring CHs based on a widely used anatomical label, LBPA40 (Shattuck et al., 2008), to form an ROI. All defined CHs were included in one of the ROIs. There were 10 ROIs in the left hemisphere and 9 in the right hemisphere (Table 2). We computed the mean activity using the average oxyHb concentration (Session 1) and *t* values (Session 2) across CHs within the same ROIs. This method was employed in previous fNIRS studies (Yanagisawa et al., 2010; Byun et al., 2014).

**Table 2.** CH coordination and regions of measurement

Anatomical structures	CH	MNI coordinates			Anatomical structures	ch	MNI coordinates		
		*x*	*y*	*z*			*x*	*y*	*z*
L middle frontal gyrus	1	−39	1	64	R middle frontal gyrus	31	48	12	55
	4	−46	12	55	R PreCG	27	40	2	64
L PreCG	8	−55	−3	51		34	57	−4	51
	11	−60	8	35		38	62	6	36
	18	−64	5	10	R postcentral gyrus	26	40	−23	71
L postcentral gyrus	2	−39	−23	71		30	49	−14	62
	5	−47	−14	61		41	69	−8	29
	12	−63	−17	44		45	66	2	9
	15	−67	−8	28	R STG	44	71	−23	17
L STG	19	−68	−21	17		48	71	−11	−3
	20	−68	−48	18	R MTG	43	68	−50	19
	22	−67	−9	−3		47	73	−37	3
L MTG	23	−71	−35	2	R inferior temporal gyrus	46	63	−64	0
	24	−64	−61	0	R superior parietal gyrus	25	34	−47	72
L superior parietal gyrus	3	−35	−46	71		29	47	−38	64
	6	−46	−37	64	R supramarginal gyrus	33	57	−28	57
L supramarginal gyrus	9	−56	−27	56		37	65	−19	45
	13	−62	−44	46		40	69	−35	34
	16	−68	−34	32	R angular gyrus	28	38	−62	62
L angular gyrus	7	−40	−61	61		32	52	−54	57
	10	−52	−53	56		35	51	−71	41
	14	−54	−69	40		36	63	−45	47
	17	−62	−59	30		39	61	−61	31
L MOG	21	−58	−72	11	R MOG	42	55	−75	12

We also investigated the relationship between the subjective sense of body ownership in Session 1 and brain activity related to resolving discrepancies in Session 2 using Spearman's rank correlation analysis between questionnaire scores and fNIRS data.

*p* values were corrected with false discovery rate (FDR) control using the Benjamini–Hochberg correction (Benjamini and Hochberg, 1995) based on the number of ROIs for all fNIRS data analyses.

## Results

### Questionnaire

**Figure 3.**
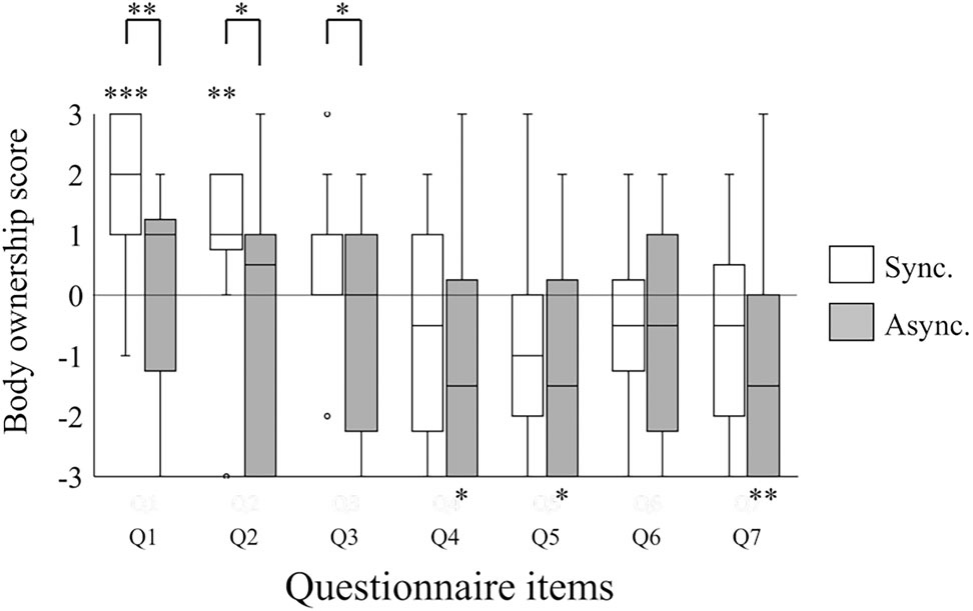
Scores for the questions on the sense of body ownership. Questionnaire items are provided in Table 1. Asterisks across the conditions indicate significant differences in between-condition comparisons, while asterisks above each condition represent significant differences from zero in one-sample tests. ***p < 0.001; **p < 0.01; *p < 0.05; Sync, synchronous condition; Async, asynchronous condition.

Comparisons between the synchronous and asynchronous conditions revealed significant differences in questionnaire Items 1, 2, and 3 (Item 1, *Z* = 2.78; *p* = 0.005; *r* = 0.66; Item 2, *Z* = 2.32; *p* = 0.020; *r* = 0.55; Item 3, *Z* = 2.535; *p* = 0.011; *r* = 0.60; median, Sync, Q1, 2.00; Q2, 1.00; Q3, 1.00; Async, Q1, 1.00; Q2, 0.50; Q3, 0.00; Fig. 3). No significant differences were found for dummy items 4–7 (*p* > 0.1). As a result of a one-sample Wilcoxon test against zero, in the synchronous condition, the scores of Items 1 and 2 were significantly higher than 0 (Item 1, *Z* = 3.87; *p* < 0.001; *r* = 0.91; Item 2, *Z* = 2.68; *p* = 0.007; *r* = 0.63). The score for Item 3 was not significant (*Z* = 1.42; *p* = 0.156; *r* = 0.33). No significant results were found for the asynchronous condition (Item 1, *Z* = 0.28; *p* = 0.777; *r* = 0.07; Item 2, *Z* = 1.01; *p* = 0.311; *r* = 0.24; Item 3, *Z* = 1.84; *p* = 0.066; *r* = 0.43). These results indicate that the synchronous, but not the asynchronous, condition elicited the sense of body ownership toward the avatar. Four dummy items showed no significant differences (Item 4, *Z* = 0.64; *p* = 0.523; *r* = 0.15; Item 5, *Z* = 0.39; *p* = 0.695; *r* = 0.09; Item 6, *Z* = 0.17; *p* = 0.863; *r* = 0.04; Item 7, *Z* = 1.53; *p* = 0.125; *r* = 0.36; median, Sync, Q4, −0.50; Q5, −1.00; Q6, −0.50; Q7, −0.50; Async, Q4, −1.50; Q5, −1.50; Q6, −0.50; Q7, −1.50). As a result of a one-sample Wilcoxon test against zero, in the asynchronous condition, the scores of Items 4, 5, and 7 were significantly lower than 0 (Item 4, *Z* = 2.01; *p* < 0.045; *r* = 0.47; Item 5, *Z* = 2.32; *p* = 0.020; *r* = 0.55; Item 7, *Z* = 2.65; *p* = 0.008; *r* = 0.62).

### NIRS data

#### Session 1

**Figure 4.**
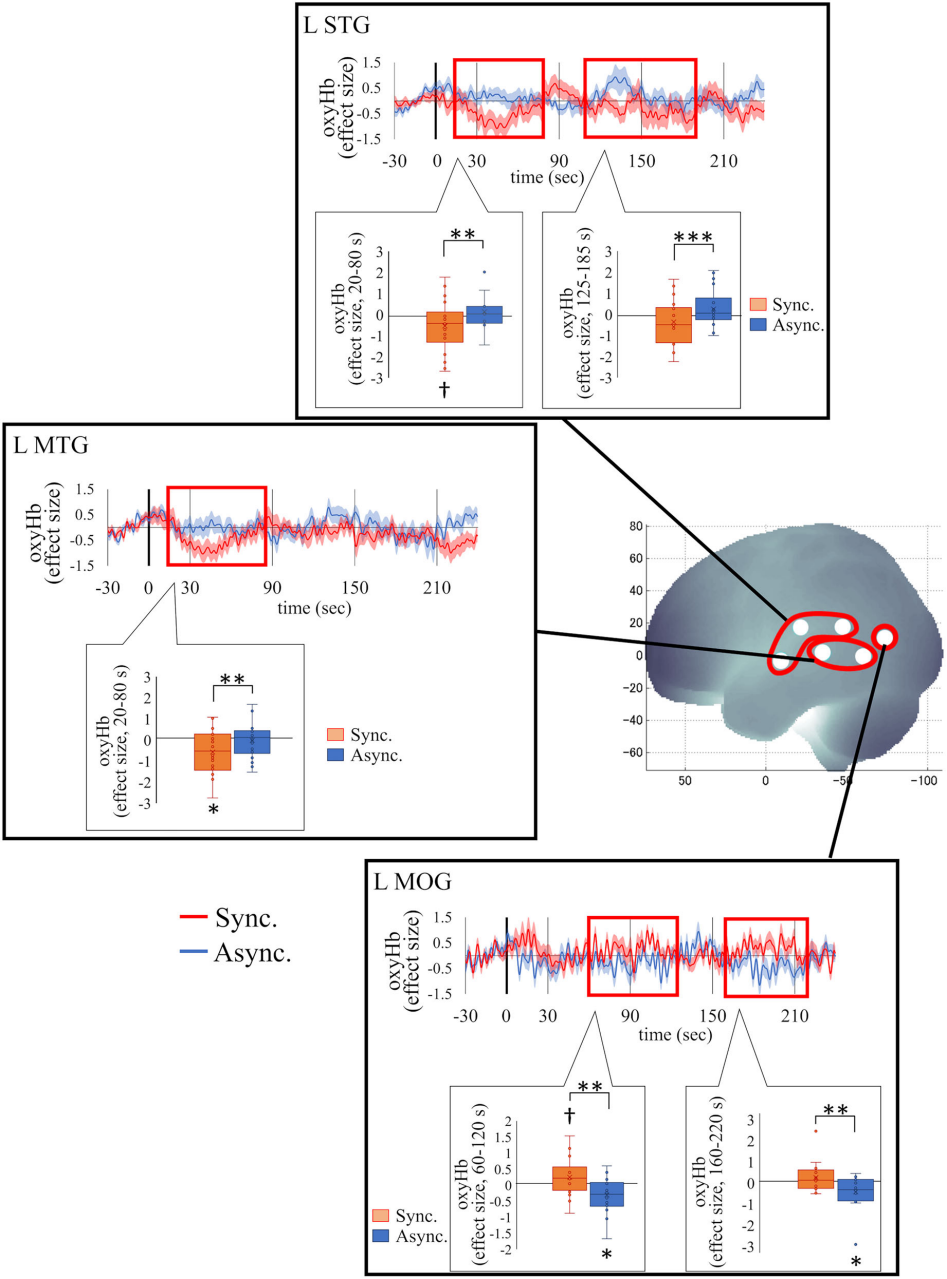
Changes and differences between the synchronous and asynchronous conditions in the oxyHb effect size in the L STG, L MTG, and L MOG during Session 1. In the L STG, oxyHb (effect size) in the synchronous condition was significantly lower than in the asynchronous condition during the periods from 15 to 80 s and from 110 to 190 s and in the L MTG from 15 to 85 s. In contrast, oxyHb in the synchronous condition was significantly greater than in the asynchronous condition during the periods from 60 to 125 s and from 160 to 220 s in the L MOG. The shaded areas represent the standard error. The areas marked with a red box indicate periods where significant differences between conditions were observed. Asterisks above or under each condition represent significant difference from zero in one-sample tests. STG, superior temporal gyrus; MTG, middle temporal gyrus; MOG, middle occipital gyrus. ***p < 0.001; **p < 0.01; *p < 0.05; ^†^ < 0.10.

**Figure 5.**
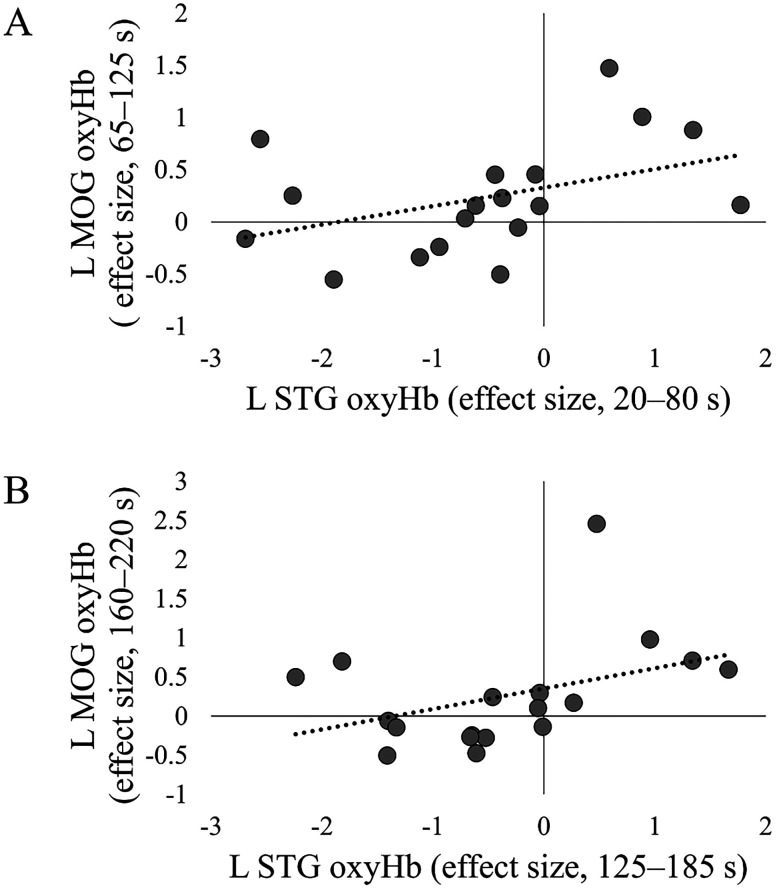
Significant positive correlations between oxyHb effect sizes in the L STG and L MOG in Session 1. ***A***, The OxyHb effect size in the left STG during 20–80 s was positively correlated with that in the left MOG during 65–125 s. ***B***, The OxyHb effect size in the left STG during 125–185 s was positively correlated with that in the left MOG during 160–220 s.

First, the results from the full-body illusion induction phase (Session 1; Fig. 4) are presented, including the duration of significant differences, the *p* value, and the effect size (*Z* and *r* values). If several successive moving windows were significant, the ranges for *p*, *Z*, and *r* values were provided. Activity in the left middle occipital gyrus (L MOG, MNI, *x* = −58; *y* = −72; *z* = 11), a region near the coordinates identified as the EBA in previous studies (Gentile et al., 2013, *x* = −48; *y* = −66; *z* = 2; Limanowski et al., 2014, *x* = −42; *y* = −68; *z* = 8; Limanowski and Blankenburg, 2016, *x* = −48; *y* = −74; *z* = 6), was significantly higher in the synchronous condition compared with the asynchronous condition (60–125 s, *Z*, 2.89–2.95; *p*, 0.003–0.004; *r*, 0.68–0.70; 160–220 s, *Z* = 2.83; *p* = 0.005; *r* = 0.67, FDR-corrected). A one-sample test revealed a trend of increased activity in L MOG in the synchronous condition (70–130 s, *Z* = 1.65; *p* = 0.099; *r* = 0.39), whereas significant deactivations in the asynchronous condition were observed (60–125 s, *Z* = 2.07; *p* = 0.038; *r* = 0.49; 155–225 s, *Z*, 2.06–2.31; *p*, 0.021–0.038; *r*, 0.49–0.54). In contrast, activity in the left superior temporal gyrus (L STG) and middle temporal gyrus (L MTG) was significantly lower in the synchronous condition than in the asynchronous condition (L STG, 15–80 s, *Z*, 2.66–2.77; *p*, 0.006–0.008; *r*, 0.63–0.65; 110–190 s, *Z*, 3.21–3.79; *p*, 0.0002–0.001; *r*, 0.76–0.89; L MTG, 15–85 s, *Z*, 2.83–3.27; *p*, 0.001–0.005; *r*, 0.67–0.77). Deactivation in the L STG (10–80 s, *Z*, 1.65–1.83; *p*, 0.074–0.099; *r*, 0.39–0.43) and L MTG (15–75 s, *Z* = 2.02; *p* = 0.043; *r* = 0.48; 175–235 s, *Z* = 2.07; *p* = 0.038; *r* = 0.49) in the synchronous condition was confirmed. To investigate the relationship between the L STG/MTG and the L MOG in the occurrence of the full-body illusion, we conducted post hoc correlation analyses under the synchronous condition. Correlations were calculated between time windows in each region that showed significant differences between conditions. As a result, a significant positive correlation was found between activity in the L STG from 20 to 80 s and in the L MOG from 65 to 125 s (*r* = 0.47; *p* = 0.050) and between the L STG from 125 to 185 s and the L MOG from 160 to 220 s (*r* = 0.49; *p* = 0.043; Fig. 5). The activity of the L MTG did not correlate with that of the L MOG. These results indicate that participants with a more deactivated L STG exhibit lower subsequent activity in the L MOG.

**Figure 6.**
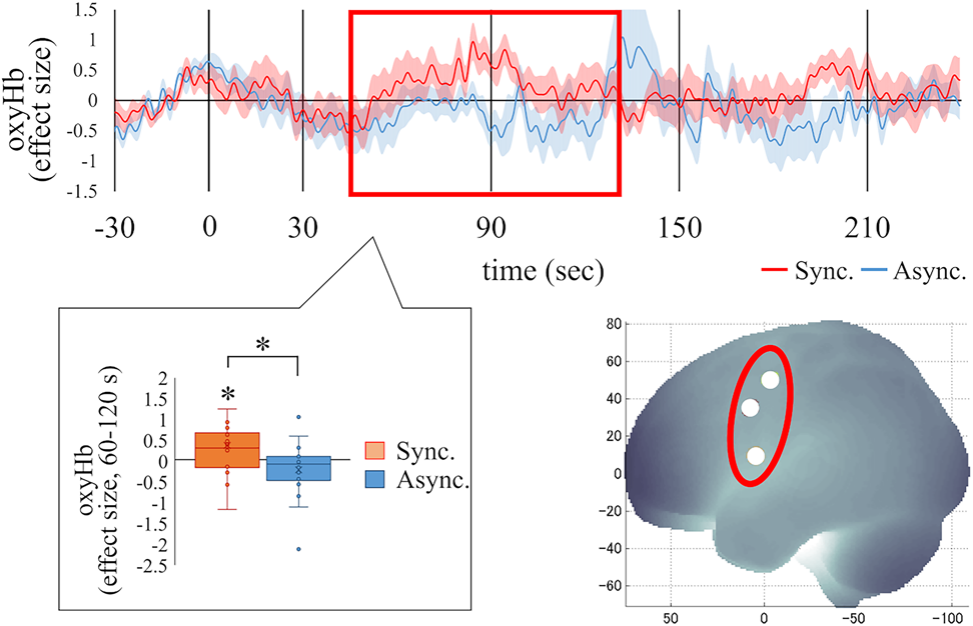
Changes and differences between the synchronous and asynchronous conditions in the oxyHb effect size in the L PreCG during Session 1. In the L PreCG, oxyHb (effect size) in the synchronous condition was significantly greater than in the asynchronous condition during the periods from 45 to 130 s (uncorrected). The shaded areas represent the standard error. The areas marked with a red box indicate periods where significant differences between conditions were observed. Asterisks above or under each condition in the boxplot represent a significant difference from zero in one-sample tests. PreCG, precentral gyrus. *p < 0.05 (uncorrected).

Additionally, the results for the precentral gyrus (PreCG), a region likely corresponding to the premotor cortex, are presented (Fig. 6). Although activations in the PreCG did not survive FDR correction, significant higher activations in the left PreCG in the synchronous condition than in the asynchronous condition were observed (45–130 s, *Z*, 2.01–2.27; *p*, 0.023–0.045; *r*, 0.47–0.53; uncorrected). A one-sample test revealed significant activations in the synchronous condition (60–130 s, *Z* = 2.07; *p* = 0.038; *r* = 0.49).

#### Session 2

**Figure 7.**
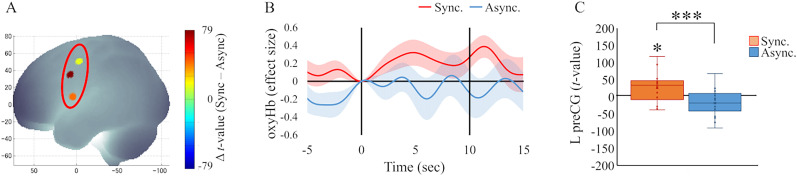
Brain activity in Session 2. ***A***, The PreCG showed a significant difference between the synchronous and asynchronous conditions. ***B***, Average changes in oxyHb (effect size) across the three stroke trials in the L PreCG. The shaded areas represent the standard error. ***C***, The *t* value (GLM analysis) in the synchronous condition was significantly higher than in the asynchronous condition. PreCG, precentral gyrus; ***p < 0.001; *p < 0.05.

The results of the analysis of brain activity induced by the visuotactile discrepancy (no tactile stimuli, only visual stimuli) during Session 2 are presented below. Brain activity in the synchronous condition was significantly higher than in the asynchronous condition in the left PreCG (*Z* = 3.34; *p* < 0.001; *r* = 0.79; FDR-corrected; Fig. 7). Increased activity in the L PreCG in the synchronous condition was also confirmed by a one-sample test (*Z* = 2.41; *p* = 0.016; *r* = 0.57). These findings suggest that the stimulus discrepancy following the occurrence of the full-body illusion induced L PreCG activity.

**Figure 8.**
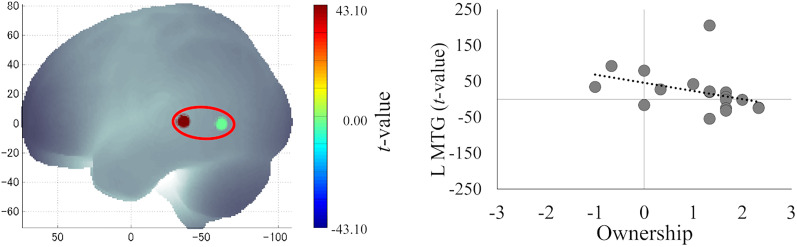
Significant negative correlation between brain activity in Session 2 (*t* value) in the L MTG and the strength of subjective body ownership.

Additionally, correlation analysis between brain activity and the subjective strength of the sense of body ownership revealed a significant negative correlation in the L MTG in the synchronous condition (*r* = −0.63; *p* = 0.005; FDR-corrected; Fig. 8), which was the same region where deactivation was observed in Session 1 (Fig. 4). This negative correlation indicates that higher L MTG activity during the stimulus discrepancy in Session 2 was associated with a weaker sense of body ownership in Session 1. No significant correlations were observed in the asynchronous condition.

## Discussion

In this study, we investigated the neural mechanisms underlying the full-body illusion by measuring brain activity during a VR-based, third-person perspective full-body illusion using fNIRS. In Session 1, we found that the induction of the full-body illusion involved deactivation in the superior and middle temporal gyri, including the STS and TPJ. This was followed by higher activity in the synchronous condition (marginal activation) compared with the asynchronous condition (significant deactivation) in the L MOG (likely EBA). In Session 2, following the induction of the illusion, the introduction of visuotactile discrepancies elicited activity in the premotor cortex. Additionally, in the same temporal lobe region as in Session 1, deactivation correlated with body ownership scores. These findings provide novel insights into the distinct roles of the premotor and occipitotemporal cortices in the full-body illusion, highlighting their contributions to the neural basis of body ownership.

### Brain activity during the induction of the full-body illusion

The left superior and middle temporal gyri and the L MOG showed significant differences in brain activity between the conditions in Session 1. The significant deactivation in the superior and middle temporal gyri during the early period (15–80 s from onset) of the stimulation phase is likely related to the function of those areas in recognizing others. The superior temporal sulcus (STS), located between the STG and MTG, is known to be involved in processing biological motion and facial and postural features of others (Allison et al., 2000; Yovel and O’Toole, 2016). Deactivation in the left superior and middle temporal gyri in the synchronous condition likely reflected suppression of the function of the STS in recognizing others. Suppression of processing the avatar as “other” would likely facilitate incorporation of the avatar's body as the participant's “own” body. Conversely, the significant activation observed after 110 s from onset in the asynchronous condition reflects recognition of the avatar as another (not the participant themself). Notably, deactivation of the STG during the rubber hand illusion has also been reported in previous research (Abdulkarim et al., 2023).

The STG defined in this study includes the TPJ, which has been implicated in self-location coding in previous research on the full-body illusion (Ionta et al., 2011, 2014; Blanke et al., 2015). In a previous study (Ionta et al., 2011), participants lying in an MRI scanner viewed an avatar positioned above them while their back and the avatar's back were stroked synchronously. Interestingly, approximately half of the participants (the “up-group”) perceived the avatar as being above them and reported a corresponding sensation of their own body moving upward during the illusion. In contrast, the remaining participants (the “down-group”) perceived the avatar as below them and experienced their body as moving downward. During this process, TPJ activity (*x* = −54; *y* = −32; *z* = 20) significantly decreased in the up-group but increased in the down-group. In our experiment, using fNIRS allowed us to conduct the study in an upright posture rather than in the supine position required for fMRI. The observed TPJ deactivation under synchronous conditions in our study (see Table 2) is similar to the activity pattern of the up-group reported by Ionta et al. Although shifts in self-location (Ehrsson, 2007; Lenggenhager et al., 2007) were not measured, the findings partially support previous results and provide insights into the relationship between the TPJ and self-location.

The greater activity in the synchronous condition compared with the asynchronous condition, along with the significant deactivation in the asynchronous condition in the L MOG, likely reflects the activity of the left EBA. Previous studies using fMRI have found activity in the left occipital gyrus during the rubber hand illusion (Gentile et al., 2013; Limanowski et al., 2014; Limanowski and Blankenburg, 2016). Some of these studies confirmed that occipital gyrus activity reflected left EBA activity by using a localizer task presenting body and nonbody images (Limanowski et al., 2014, *x* = −42; *y* = −68; *z* = 8; Limanowski and Blankenburg, 2016, *x* = −48; *y* = −74; *z* = 6). The coordinates of the L MOG in our study (*x* = −51; *y* = −72; *z* = 11) are close to those reported in previous studies, and it is plausible that the activity of the L MOG in our study reflects the activity of the EBA. The EBA, together with the PMv and parietal cortex—both of which were highlighted in early research on body ownership (Ehrsson et al., 2004; Ehrsson, 2012; Blanke et al., 2015)—and subsequently identified regions such as the cerebellum and putamen (Ehrsson, 2020) form an important network for the sense of body ownership. Several studies argue that the left EBA, left PMv, and left parietal lobe, in particular, play crucial roles in this network (Wold et al., 2014; Limanowski and Blankenburg, 2015, 2016). For example, it has been reported that rTMS applied to the left EBA increased proprioceptive drift in the rubber hand illusion, suggesting that the stimulation likely disturbed the integration of visual and proprioceptive information (Wold et al., 2014). Furthermore, the left EBA has been shown to be active during the rubber hand illusion (Gentile et al., 2013; Limanowski and Blankenburg, 2015, 2016), and its activity positively correlates with the subjective strength of the illusion (Limanowski et al., 2014). It has also been reported that mental imagery involving the transformation of the location of the participant's body toward the body image presented on the screen in front of them activated the left EBA (Arzy et al., 2006). Beyond these studies, activity in the EBA has also been reported during first-person full–body illusions (Gentile et al., 2015; Guterstam et al., 2015b; Preston and Ehrsson, 2016), although these studies did not specifically focus on the functional role of the EBA, thereby providing limited explanation regarding its involvement in the illusion. In our experiment as well, the EBA showed a marginally significant increase in activity during the illusion. In contrast, the deactivation of the left EBA in the asynchronous condition likely reflects inhibition of the recognition of the avatar as one's own. Collectively, these findings suggest that the EBA plays an important role in the emergence of the sense of body ownership, particularly in integrating visual, somatosensory, and proprioceptive information.

A positive correlation was observed between the activity of the STG and that of the EBA. This result suggests that participants who exhibited greater suppression of L STG activity subsequently showed lower activation in the EBA during the induction of the illusion. This finding suggests that effective suppression of other-body representation reduces the demand for multisensory integration in the EBA. Conversely, when the suppression of other-body representation in the STS is less effective, the EBA may compensate by increasing its activity to generate the sense of body ownership.

As in previous studies, premotor activity was observed during the full-body illusion in our experiment, though this activity did not survive FDR correction. The relatively lower activation of the premotor cortex in Session 1 is likely due to the third-person perspective. In third-person perspective illusions, the subjective strength of the illusion is weaker (Maselli and Slater, 2013, 2014; Preston et al., 2015; Gorisse et al., 2017), and activation in the premotor cortex is also reduced (Petkova et al., 2011a). Additionally, Ionta et al. (2011), using a third-person perspective paradigm, found no premotor activation. A more detailed interpretation of premotor activity is discussed in the following section.

### Brain responses to the visuotactile discrepancy after full-body illusion occurrence

Our results showed that when only visual stimuli were presented in Session 2, after the full-body illusion was elicited in Session 1, the left PreCG was more activated than when the full-body illusion was not elicited. Activation of the PreCG has been frequently reported in studies on the sense of body ownership, and the premotor cortex in this region is thought to be involved in the integration of multisensory signals (Ehrsson et al., 2004; Petkova et al., 2011a; Gentile et al., 2013; Limanowski and Blankenburg, 2015; Gentile et al., 2015; Guterstam et al., 2015a,b). In addition, recent studies have suggested that the premotor cortex updates body representation by continuously integrating existing internal bodily representations and multisensory signals (Limanowski, 2022; Castro et al., 2023). Specifically, it has been proposed that the premotor cortex contributes to estimating the sources of multisensory signals based on internal representations (Fang et al., 2019), as well as to the ongoing construction of these internal representations (Guterstam et al., 2019). Since only visual stimuli were presented in Session 2, the activation is likely a response to the discrepancy.

However, premotor activity was observed only in the synchronous condition in Session 2, while it was absent in the asynchronous condition. This is important, as it suggests that premotor activity is not merely a response to detecting multisensory discrepancies. Rather, it reflects the detection, and likely the reconciliation, of discrepancies related to the body already embodied as one's own. In the rubber hand illusion, a phenomenon known as “back-projection” has been postulated (Shimada, 2022). This refers to the transfer of changes made to the illusory rubber hand back onto the participant's hand (Kanaya et al., 2012; Osumi et al., 2014). Such a response does not occur when changes are applied to a rubber hand that has not been embodied. For example, when the rubber hand spontaneously performs a movement, such as suddenly spreading its palm, after the illusion has been induced, the participant's hand is also involuntarily drawn to perform the same movement (Shibuya et al., 2018). Furthermore, motor cortex activity during this phenomenon, as indexed by μ-suppression, significantly correlates with the body ownership score for the rubber hand. This response is not merely imitation, as it was absent in the asynchronous condition, where the rubber hand illusion did not occur. Therefore, unless projection of self-body representation onto the rubber hand occurs, back-projection does not manifest. The results, occurring only when the illusion was successfully induced in the synchronous condition, likely represent a form of back-projection, reflecting an adjustment in internal body processing to mediate the sensory discrepancies. In other words, our findings suggest that back-projection also occurs in the full-body illusion.

Predictive coding models and the free-energy principle have been used to explain self-body recognition (Limanowski and Blankenburg, 2013; Apps and Tsakiris, 2014; Seth and Tsakiris, 2018). These models propose that beliefs about the self (“self” prior) guide predictions about sensory input, which are compared with actual input and updated through prediction errors. In Session 2, the “self” prior—formed in Session 1 as the belief that the avatar was one's own body—likely generated expectations of multisensory input. When only visual input was presented without corresponding tactile stimulation, prediction errors may have arisen, prompting an update of the self-related belief. The observed activation in the premotor cortex may reflect these processes, involving the generation of sensory predictions from prior beliefs, comparison of predicted and actual inputs, and belief updating in response to prediction errors. Taken together, we consider that the function of the premotor cortex is to generate and maintain the sense of ownership by integrating internal body representations with associated external multisensory inputs.

A negative correlation was observed between the subjective strength of the illusion in Session 1 and MTG activity in Session 2. This may reflect a process in which participants who experienced weaker body ownership toward the avatar in Session 1 attempted to dissociate the self from the avatar and recognize it as an “other” to resolve the conflict in their body image caused by the visuotactile discrepancy in Session 2. Previous studies have suggested that the STS is involved in the recognition of others’ bodies (Allison et al., 2000; Yovel and O’Toole, 2016), and the MTG activity observed in this study is considered to reflect this STS function.

### Limitation

Lastly, we discuss the limitations of this study. One limitation of this study is that the experiment was conducted from a third-person perspective. Therefore, the findings may not be directly applicable to full-body illusions induced from a first-person perspective. Previous studies have reported that third-person full–body illusions are subjectively weaker than first-person illusions (Petkova et al., 2011b; Maselli and Slater, 2013, 2014; Preston et al., 2015; Gorisse et al., 2017). Additionally, Petkova et al. (2011a) found that the bilateral premotor cortex and the left intraparietal sulcus were activated during the induction of first-person perspective illusions. In contrast, Ionta et al. (2011), who employed a third-person full–body illusion paradigm, did not report activation in the premotor cortex. Petkova et al. (2011a) speculated that more efficient multisensory integration in the premotor cortex under a first-person perspective might account for the stronger sense of body ownership in a first-person full–body illusion; however, the precise mechanisms remain unclear. Our findings demonstrate that the premotor cortex is also activated during third-person full–body illusions, providing novel insights into the neural mechanisms underlying body ownership across different visual perspectives. Additionally, full-body illusions differ from rubber hand illusions in that they require the integration of individual body parts into a coherent whole-body representation (Petkova et al., 2011a; Gentile et al., 2015).

A second limitation concerns the possibility that the observed neural activities reflect not the sense of body ownership per se but rather the referral of touch (RoT; Pavani et al., 2000). In previous studies, questionnaire items related to RoT have often been analyzed as indicators of body ownership. However, Tosi et al. (2024) suggested that RoT and the sense of body ownership are distinct constructs. Given that the premotor cortex is crucial for multisensory integration, it is plausible that the premotor activity observed in this experiment could be attributed to RoT. On the other hand, it has been reported that RoT frequently co-occurs with the sense of body ownership, that RoT ratings show a positive correlation with ownership ratings, and that RoT tends to occur prior to the emergence of the sense of body ownership (Reader et al., 2021). These findings suggest that RoT may be subsumed under the sense of body ownership or that RoT may represent an initial stage in the development of body ownership. The relationship between RoT and the sense of body ownership remains an important issue for future investigation.

Another limitation of this study concerns potential confounding effects of participant movement on the fNIRS signals, particularly in motor-related regions. Although participants were instructed to remain still and were continuously monitored by the experimenter, motion tracking data were not recorded. To address this, we compared the systemic components, which reflect physiological signals such as cardiac pulsation and body motion, extracted using the same hemodynamic modality separation method (Yamada et al., 2012) employed in the main analysis. Treated as an indirect indicator of movement, this analysis revealed no significant differences between conditions (all *p* > 0.1), suggesting that the observed neural activity is unlikely to reflect movement artifacts. Nonetheless, subtle unmeasured movements may still have influenced the data. Future studies should include motion tracking as nuisance regressors to better control for movement-related confounds.

### Conclusion

This study aimed to investigate brain activity, especially in the premotor and occipitotemporal cortices, during a VR-based full–body illusion using fNIRS. The results showed that during the full-body illusion (Session 1), deactivation was observed in the left superior and middle temporal gyri, including STS and TPJ, and greater activity in the synchronous condition compared with the asynchronous condition was observed in the L MOG (EBA). The deactivation of the left STS is thought to reflect the suppression of functions related to the recognition of others, whereas the higher activation of the left EBA in the synchronous condition likely reflects the embodiment of the avatar. Its deactivation in the asynchronous condition is interpreted as a suppression of the representation of the avatar as one's own body. These findings suggest that the suppression of other-body recognition and the embodiment of the avatar's body are important for the occurrence of the sense of body ownership in the full-body illusion. In Session 2, the visuotactile discrepancy following the illusion induced activity in the left premotor cortex. In addition, suppression in the MTG, which correlated with the subjective body ownership score, was also observed. These responses may reflect the process of reconciling sensory discrepancies to maintain body ownership based on the “self” prior established earlier in Session 1. Our study provides novel insight into the distinct roles of the premotor and occipitotemporal cortices underlying the sense of body ownership in third-person full–body illusions.

## Synthesis

Reviewing Editor: Christine Portfors, Washington State University Decisions are customarily a result of the Reviewing Editor and the peer reviewers coming together and discussing their recommendations until a consensus is reached. When revisions are invited, a fact-based synthesis statement explaining their decision and outlining what is needed to prepare a revision will be listed below. The following reviewer(s) agreed to reveal their identity: NONE.

Thank you for thoroughly addressing the previous reviews. There are only minor comments remaining.

Could you please clarify description of Session 2. You say that "Session 2 examined brain activity in response to temporally incongruent visual and tactile stimuli", but also "In Session 2, only the visual stimulation of stroking the avatar's back was presented, without tactile stroking stimulation to the participant's back." Assuming no tactile stimulation at all, it was unclear what the "synchronous" and "asynchronous" conditions were here - or was there a separate "Session 2" after each, the synchronous and the asynchronous condition in Session 1?

Minor suggestions: The Discussion could be shortened a bit in parts (e.g., the bit on predictive coding on pp.21f.); and the language could be polished a bit; there are some grammatical errors here and there.
